# Probiotics in Critically Ill Patients

**DOI:** 10.5812/kowsar.22287523.2291

**Published:** 2011-09-26

**Authors:** Omid Moradi Moghaddam

**Affiliations:** 1 Department of Anesthesiology and Critical Care, Rasoul-Akram Medical Center, Tehran University of Medical Sciences, Tehran, Iran

**Keywords:** Probiotic, Intensive care unit, Patients

The term ‘probiotic’ is derived from the Greek/Latin word “pro” and the Greek word “bios,” meaning “of life”. The concept of probiotics was first described by Metchnikoff in 1907 (1). The World Health Organization (WHO) and the Food and Agriculture Organization of the United Nations have defined probiotics as “live microorganisms, which, when administered in adequate amounts, confer a health benefit on the host” (1, 2). These agents are often administered concurrently with substances that promote bacterial colonization and growth (prebiotics); in this instance, they are referred to as synbiotics.

Probiotics are bacteria or yeasts. Most probiotics come from two groups: Lactobacillus and Bifidobacterium. Each group comprises different species and strains. Some probiotics, such as Saccharomyces boulardi, are yeasts (3, 4).

In a healthy individual, there is a homeostasis between commensal intestinal microbiota and pathogenic bacteria. The composition of colonic microbiota is fundamental to gut barrier function. Anaerobic bacteria are the predominant microorganisms in the gastrointestinal tract, preventing the overgrowth of pathogenic bacteria in a phenomenon termed “colonization resistance” (2). When this homeostatic mechanism is altered, an ecological imbalance occurs, and gut barrier function is impaired. Gut barrier and immune dysfunction is associated with the onset of systemic inflammatory response syndrome (SIRS) and multiple organ dysfunction syndrome (MODS) in critically ill patients (5).

Several mechanisms by which probiotics exert their beneficial activity have been proposed, including competitive exclusion of bacterial adherence; release of bacteriocins to inhibit the growth of pathogens; production of butyrate; antioxidative effects; stimulation of mucus and SIgA production; enhancement of macromolecular degradation, which reduces antigen load; suppression of immune cell proliferation; inhibition of epithelial cell nuclear factor kappa B (NFκB) activation; modulation of epithelial apoptosis; maintenance of epithelial barrier; and modulation of immune function (2, 5, 6). There is substantial evidence that during periods of critical illness, significant alterations occur in the gut microflora due to several factors, including changes in circulating stress hormones, gut ischemia, immunosuppression, the use of antibiotics, and a lack of nutrients (6, 7). ICU and surgical patients are now believed to be at high risk of bacterial translocation, resulting in exacerbation of the disease state. Intestinal microbes can have a major role in such patients.

## Antibiotic-Associated Diarrhea

Antibiotic-associated diarrhea (AAD) is an important cause of morbidity and mortality in critically ill patients. Prevention of AAD is one of the most extensively investigated areas of probiotics. Probiotics mediate the prevention or relapse of AAD-Saccharomyces boulardi has been shown to reduce the incidence of AAD that is caused by the bacterium Clostridium difficile (3). Probiotics do not conclusively reduce the incidence of enteral tube feeding diarrhea (ETF)-related diarrhea in critically ill patients (2). The increased incidence of mortality in probiotic patients should lead to cautious use probiotic in critically ill patients. Although there have been reports that lactobacilli has benefits in infectious diarrhea, use of this probiotic is not advised in immunosuppressed or critically ill patients, because these populations are at increased risk of developing infections due to lactobacilli (8-10). Further, in vivo and animal studies are required to confirm the mortality findings in severely and critically ill patients (11). Only then can probiotics be administered safely to critically ill patients to manage enteral tube feeding-related diarrhea. In conclusion, as clinicians, we need to challenge the hypothesis that probiotics are beneficial to the critical ill patient, because the evidence to support probiotic use in the management of ETF diarrhea in critically ill patients remains unclear.

## Ventilator-Associated Pneumonia

Administering probiotics has been advocated as a means to prevent various infections, including Ventilator-associated pneumonia (VAP), in the ICU. The putative mechanism of probiotics against VAP is competition with pathogens, decreasing their density in the oropharynx and stomach of patients undergoing mechanical ventilation (MV) (9). The efficacy of probiotics against infections might be better explained by their immunomodulatory properties (5). Further research is necessary in this area, considering the increasing problem of antimicrobial resistance. Active surveillance for probiotic-induced diseases is equally important.

## Acute Pancreatitis

Approximately 20% of patients with acute pancreatitis will develop necrotizing pancreatitis. This subset of patients has a mortality rate of 10% to 30% (3), which is primarily attributable to infectious complications. These infections are believed to occur as a result of small bowel bacterial overgrowth, mucosal barrier failure, and translocation of intestinal organisms. Probiotic prophylaxis in patients undergoing abdominal operations and pancreatitis reduces infectious complications.

## Respiratory Tract Infections

The majority of randomized controlled trials indicates that the incidence of Respiratory tract infections (RTIs) is not inﬂuenced considerably by prophylactic administration of probiotics, although probiotics can reduce the severity and duration of subsequent RTIs. Probiotic use is associated with several adverse events, all of which are mild (12). Because probiotic organisms have variable effects, as has been seen in other types of human disease, further research is needed to determine the value of certain probiotic preparations in the prevention of RTIs.

## Nosocomial Pneumonia

Probiotic products reduce pathogenic colonization of the host. Despite the theoretical effects, there is insufficient evidence that probiotic products lower the incidence of nosocomial pneumonia (7). Large, multicenter, randomized clinical trials that use a rigorous, invasive diagnostic approach for nosocomial pneumonia need to be performed to prospectively evaluate the value of probiotic products. In addition, bench research needs to be performed to select the most appropriate probiotic formulation for various clinical applications. Recent clinical trial results on the use of probiotics in critically ill patients are compelling and merit further study. Well-designed, large-scale, multicenter trials that follow the Consolidated Standards of Reporting Trials (CoN SIOT) guidelines are needed (3). Issues that complicate the interpretation of currentn studies include the lack of quality control and use of different species in study designs. The most significant gap in our current knowledge regarding probiotics is their mechanism(s) of action.

Gut microbiota modiﬁers include probiotics, prebiotics, and synbiotics. Current evidence suggests that probiotics enhance the efﬁcacy of prebiotic supplementation; thus, they should be given in combination. There is increasing awareness of the potential beneﬁts of prebiotic supplementation, and a growing body of data suggests their value in the ICU. The 2007 update of the Canadian Guidelines concluded that there are insufﬁcient data to make a recommendation on the use of prebiotics/probiotics/synbiotics in critically ill patients. Similarly, the recent Guidelines on Enteral Nutrition from the European Society of Parenteral and Enteral Nutrition (ESPEN) recommend that “a combination of different ﬁbers, probiotics and prebiotics should be studied because of synergistic effects in different diseases” (5). For the future, researchers must identify the best type, the best combination of ﬁbers for enteral nutrition, or both in the ICU. Further, a combination of ﬁbers, prebiotics, and probiotics should be studied, because additive or synergistic effects in various acute and critical situations can exist. However, in critically ill patients with a high risk of nonocclusive intestinal ischemia and in those with predicted SAP, synbiotics can be harmful and are not recommended.

There is, however, not enough evidence to justify their routine use for the prevention of nosocomial infections, including hospital-acquired pneumonia. In addition, they can behave as pathogens in immunocompromised hosts. Bench research needs to be performed to identify strain-specific qualities that could guide species selection for particular clinical problems. Because we do not expect all antibiotics to be useful for all conditions, we should not expect all probiotics to be equal and effective for every condition. It is likely that as our knowledge grows regarding these organisms, broad-spectrum and selective probiotic strains will be developed for speciﬁc disease conditions.
